# Individual and household risk factors for Ebola disease among household contacts in Mubende and Kassanda districts, Uganda, 2022

**DOI:** 10.1186/s12879-024-09439-1

**Published:** 2024-05-30

**Authors:** Stella M. Migamba, Denis-Luc Ardiet, Richard Migisha, Hildah T. Nansikombi, Brian Agaba, Helen Nelly Naiga, Mercy Wanyana, Jane Frances Zalwango, Immaculate Atuhaire, Peter Chris Kawungezi, Marie Goretti Zalwango, Brenda Simbwa, Daniel Kadobera, Alex R. Ario, Julie R. Harris

**Affiliations:** 1 Uganda Public Health Fellowship Program, Uganda National Institute of Public Health, Kampala, Uganda; 2 Department of Epidemiology and Training, Epicentre, Paris, France; 3 Division of Global Health Protection, U.S. Centers for Disease Control and Prevention, Kampala, Uganda

**Keywords:** Sudan virus disease, Ebola disease, Sudan virus, Uganda

## Abstract

**Background:**

In 2022, an Ebola disease outbreak caused by Sudan virus (SUDV) occurred in Uganda, primarily affecting Mubende and Kassanda districts. We determined risk factors for SUDV infection among household members (HHM) of cases.

**Methods:**

We conducted a case-control and retrospective cohort study in January 2023. Cases were RT-PCR-confirmed SUDV infection in residents of Mubende or Kassanda districts during the outbreak. Case-households housed a symptomatic, primary case-patient for ≥ 24 h and had ≥ 1 secondary case-patient with onset < 2 weeks after their last exposure to the primary case-patient. Control households housed a case-patient and other HHM but no secondary cases. A risk factor questionnaire was administered to the primary case-patient or another adult who lived at home while the primary case-patient was ill. We conducted a retrospective cohort study among case-household members and categorized their interactions with primary case-patients during their illnesses as none, minimal, indirect, and direct contact. We conducted logistic regression to explore associations between exposures and case-household status, and Poisson regression to identify risk factors for SUDV infection among HHM.

**Results:**

Case- and control-households had similar median sizes. Among 19 case-households and 51 control households, primary case-patient death (adjusted odds ratio [OR_adj_] = 7.6, 95% CI 1.4–41) and ≥ 2 household bedrooms (OR_adj_=0.19, 95% CI 0.056–0.71) were associated with case-household status. In the cohort of 76 case-HHM, 44 (58%) were tested for SUDV < 2 weeks from their last contact with the primary case-patient; 29 (38%) were positive. Being aged ≥ 18 years (adjusted risk ratio [aRR_adj_] = 1.9, 95%CI: 1.01–3.7) and having direct or indirect contact with the primary case-patient (aRR_adj_=3.2, 95%CI: 1.1–9.7) compared to minimal or no contact increased risk of Sudan virus disease (SVD). Access to a handwashing facility decreased risk (aRR_adj_=0.52, 95%CI: 0.31–0.88).

**Conclusion:**

Direct contact, particularly providing nursing care for and sharing sleeping space with SVD patients, increased infection risk among HHM. Risk assessments during contact tracing may provide evidence to justify closer monitoring of some HHM. Health messaging should highlight the risk of sharing sleeping spaces and providing nursing care for persons with Ebola disease symptoms and emphasize hand hygiene to aid early case identification and reduce transmission.

**Supplementary Information:**

The online version contains supplementary material available at 10.1186/s12879-024-09439-1.

## Background

Ebolaviruses have the potential to cause both small and large outbreaks. Human Ebola disease (EBOD) outbreaks typically occur after humans have contact with the body fluids or meat of infected non-human primates [1] or fruit bats [2]. Person-to-person transmission occurs through direct contact with body fluids (such as blood, saliva, urine, sweat, vomit, faeces, breast milk, semen, vaginal fluid) or tissues of an infected, symptomatic person or corpse [3]. Among household contacts, EBOD is transmitted largely through direct physical contact with a symptomatic patient [4–6]. Transmission through contaminated inanimate objects (fomites) occurs, but is less common [5, 6]. A meta-analysis of household secondary attack rates for EBOD showed that risk was highest for household members providing nursing care (48%), and lowest for household members without any direct contact (0.8%) [5].

The first and largest EBOD outbreak in Uganda, caused by Sudan virus (SUDV), occurred in 2000 and included 425 cases and 224 deaths; additional EBOD outbreaks in Uganda occurred in 2007, 2011, 2012, and 2019 [7]. On September 20, 2022, the Uganda Ministry of Health (MoH) declared an outbreak of Sudan virus disease (SVD) after a case was confirmed the previous day in a 26-year-old man living in Mubende District, Central Uganda [8, 9]. The response to the outbreak included immediate and intensive efforts to control the outbreak and stop transmission, including rapid identification, isolation, and treatment of cases, contact tracing and monitoring of contacts [10, 11]. On October 15, in response to the spread to other districts, the president of Uganda instituted a 21-day lockdown in Mubende and Kassanda districts, the epicentres of the outbreak [12].

By the end of the outbreak, there were 142 confirmed cases of SVD in nine districts in Uganda [13]. In total, 4,793 contacts had been listed and monitored [14]. Household and community transmission accounted for two-thirds of cases [15]. As persons who typically interact the most closely with EBOD patients before they reach health facilities, household members of infected persons are at high risk of exposure during an outbreak. As in previous outbreaks [3, 6], during the 2022 SUDV outbreak in Uganda, some households had multiple cases. Due to the nonspecific symptoms of early infection, appropriate precautions may not be taken by household members. EBOD symptoms are often similar to those of other diseases such as malaria or diarrhoeal diseases. Knowing which individual or household characteristics are associated with the highest risk of household transmission can provide information on tailored community education in an outbreak-affected area, and may provide information on contacts who need to be monitored especially carefully. We determined risk factors for Sudan virus (SUDV) infection among household members of confirmed cases in Mubende and Kassanda districts during the 2022 outbreak.

## Methods

### Study setting

This study was conducted in Mubende and Kassanda districts in Central Uganda, where 80% of the SUDV cases in the 2022 outbreak were identified [15]. The mid-year population projections for 2021 were 582,900 for Mubende District and 319,900 for Kassanda District [16]. Subsistence farming is the most common occupation [17].

### Study design

We conducted both a case-control study and a retrospective cohort study in January 2023.

#### Case-control study

The case-control study was designed to understand household factors that increased the odds of presumed household transmission. Case-households were homes that housed a symptomatic, confirmed case-patient for at least 24 h and had a secondary case-patient with onset < 2 weeks after their last exposure to the primary case-patient (presumed infected from the primary case-patient). Control households were homes that housed a symptomatic, confirmed case-patient for at least 24 h but did not have another person who developed an infection at home at any point. Both case- and control households had to have at least one household member (HHM) besides the primary case-patient.

We identified case and control households from the SUDV confirmed case line list and narrative notes. Eligible households for the case-control study were selected based on the following criteria: First, the primary case-patient (the first person to develop confirmed SUDV in the household) had to have spent at least 24 h at home while ill before being evacuated to the Ebola Treatment Unit (ETU). Second, the household had to have ≥ 1 HHM other than the primary case-patient who also spent at least 24 h in the home while the primary case-patient was ill and thus had a risk of becoming infected. All primary and secondary case-patients were laboratory-confirmed cases. Laboratory testing for SUDV was done at the Uganda Virus Research Institute (UVRI) laboratory, which was the designated national reference laboratory for viral haemorrhagic fever testing. SUDV infection was detected using real-time polymerase chain reaction (PCR) test [8, 13, 15].

#### Cohort study

Using only the case-households from the case-control study, we conducted a retrospective cohort study to identify individual risk factors for SUDV infection among all household members of primary case-patients. The cohort comprised all consenting case-HHM. We excluded HHM who could not be reached due to logistical reasons (relocation or not available by phone), who died without any next-of-kin available to interview, and who had mental disorders.

### Data collection and study variables

For the case-control study, we administered questionnaires [18] to either the primary case-patient, if alive, or another adult who lived in the household while the primary case-patient was ill. We collected data on the primary case-patient’s socio-demographic characteristics, presence of symptoms, number of days the primary case-patient was at home with symptoms, household location (urban or rural), number of rooms and bedrooms in the household, isolation practices for the primary case-patient, whether or not the primary case-patient had a single dedicated caretaker at home, presence of handwashing facilities, and type of care HHM provided to the primary case-patient (interactions).

For the cohort study, we collected data by interviewing every consenting adult HHM who lived in a case-household while the primary case-patient was ill, using a standardized questionnaire. For minors, we interviewed guardians, and for HHM who died, we interviewed a proxy. The objectives of these interviews were to characterise the level of interaction between household members and the primary case-patient. We collected data on HHMs’ socio-demographic characteristics, whether they had underlying conditions or not, which symptoms they developed, whether they were tested for SUDV, whether they suspected that the primary case-patient had SVD, ways in which the contact interacted with the primary case-patient after his or her onset, use of gloves, access to a handwashing facility with soap and water, access to information on the provision of safe care, and the clinical outcome of the primary case-patient.

Interactions were grouped into mutually exclusive groups to compare exposure-outcome associations against a common reference group. Interactions of the contact with the primary case-patient were categorised as no contact, minimal contact, indirect contact, and direct contact. ‘No contact’ referred to having had no interaction at all with the primary case-patient. ‘Minimal contact’ referred to having sat with or talked to the primary case-patient in the same room, or having removed dishes after meals or rode on the same motorbike, but none of the higher-level interactions. ‘Indirect contact’ included having washed the primary case-patient’s clothes, changed their beddings, or cleaned their room but none of the higher-level interactions. ‘Direct contact’ including having played with the primary case-patient or bathed, cleaned, carried, helped move around, fed or breastfed, shared dishes or utensils at meals, or shared a bed, or had sexual intercourse with the primary case-patient.

We considered every HHM who was tested for SUDV infection and received a positive result to have SVD and every other contact to be negative (including those who were not tested). None of the HHM who were not tested reported any symptoms.

### Data analysis

#### Case-control study

We conducted logistic regression to explore possible associations between each exposure variable and case-household status. Odds ratios and their associated 95% confidence intervals were used as measures of effect size. Exposures with *p*-values < 0.2 were included in the multi-variable model. Multivariable analysis was done to determine predictors of being a case household.

#### Cohort study

We fitted generalized linear models using Poisson regression analysis to identify risk factors for SUDV infection among household members to primary case-patients and adjusted for clustering at household level. We included variables as categorical fixed effects nested within fixed household identifiers and assumed a normal distribution of the random effects.

We computed risk ratios with 95% confidence intervals to determine associations between exposures and confirmed SUDV infection. Exposures with *p* values < 0.2 were evaluated in multivariable analysis after checking for collinearity of variables to determine factors independently associated with SUDV infection. Stata version 14 (StataCorp, CollegeTexas, USA) was used for statistical analysis.

## Results

### Characteristics of case- and control-households

During the outbreak, there were 84 households with ≥ 1 case-patient, of which 70 households were eligible for the case-control study (i.e., had at least one HHM besides the primary case-patient). Of these, 19 were case-households and 51 were control households. Twelve (63%) case-households and 38 (75%) control households had a primary case-patient aged ≥ 18 years. The primary case-patient in 17 (89%) case-households and 28 (55%) control households died. Case- and control households had similar median household sizes (6 HHM, IQR 4–9 for case-households and 6 HHM, IQR 4–8 for control households). Ten (67%) case-households and 23 (62%) control households had at least 6 HHM (Table 1). All households (100%) reported at least one kind of care interaction of HHM with the primary case-patient.

### Factors associated with household SUDV transmission

In multivariable analysis, households in which the primary case-patient died (whether at home or in hospital) had nearly eight times higher odds of becoming case-households than those in which the primary case-patient recovered (OR_adj_=7.6, 95% CI: 1.4–41). Households with ≥ 2 bedrooms had lower odds of being-case households than those that had only one bedroom (OR_adj_=0.19, 95% CI: 0.056–0.71) (Table 1).

**Table 1:** Characteristics of case and control households during the Ebola outbreak in Mubende and Kassanda districts, Uganda, 2022

Variable (*n* = 70)	Case HH	Control HH		
	*n* (%)	*n* (%)	cOR (95% CI)	aOR (95% CI)
**Primary case-patient sex**				
Male	12 (63)	27 (53)	1	
Female	7 (37)	24 (47)	0.66 (0.22–1.9)	
**Age of primary case-patient (yrs)**				
< 18	7 (37)	13 (25)	1	1
≥ 18	12 (63)	38 (75)	0.59 (0.19–1.8)	0.67 (0.18–2.5)
**Clinical outcome of primary case-patient**				
Recovered	2 (11)	23 (45)	1	1
Died	17 (89)	28 (55)	**6.9 (1.5–33)**	**7.6 (1.4–41)**
**District**				
Mubende	12 (63)	36 (71)	1	
Kassanda	7 (37)	15 (29)	1.4 (0.46–4.2)	
**HH location**				
Urban	6 (32)	19 (37)	1	
Rural	13 (68)	32 (63)	1.3 (0.42–3.9)	
**HH bedrooms**				
1	10 (53)	11 (22)	1	1
≥ 2	9 (47)	40 (78)	0.25 (0.081–0.76)	0.19 (0.056–0.71)
**HH rooms**				
1	4 (21)	6 (12)	1	
≥ 2	15 (79)	45 (88)	0.50 (0.12–2.01)	
Number of HHM*				
2─5	9 (47)	14 (38)	1	
≥ 6	10 (53)	23 (62)	0.67 (0.22–2.07)	
**Ratio HHM: bedrooms**				
1:1─4:1	13(68)	31 (84)	1	
> 4:1	6 (32)	6 (16)	2.4 (0.65–8.8)	
**Ratio HHM: rooms**				
1:1─2:1	12 (63)	20 (54)	1	
3:1─9:1	7 (37)	17 (46)	0.69 (0.22–2.1)	
**Household had one dedicated caretaker for primary case-patient**				
No	10 (53)	24 (47)	1	1
Yes	9 (47)	27 (53)	0.80 (0.28–2.3)	0.81 (0.24–2.7)
**Household had piped water for handwashing**				
No	17 (89)	46 (90)	1	
Yes	2 (11)	5 (10)	1.1 (0.19–6.1)	
**Household had electricity**				
No	5 (26)	12 (24)	1	
Yes	14 (74)	39 (76)	0.86 (0.26–2.9)	
**Primary case-patient had an underlying condition**				
No	16 (84)	38 (75)	1	
Yes	3 (16)	13 (25)	0.55 (0.14–2.2)	
**Days primary case-patient was ill at home before evacuation**				
1	2 (10)	5 (10)	1	1
2─4	7 (37)	18 (35)	0.97 (0.15–6.2)	1.5 (0.18-13)
≥ 5	10 (53)	28 (55)	0.89 (0.15–5.4)	1.6 (0.21-12)
**Primary case-patient stayed isolated at home during illness**				
No	18 (95)	45 (88)	1	
Yes	1 (5)	6 (12)	0.42 (0.047-3.7)	

*Of the 70 households, data on household size were only available for 56, including 19 case-households and 37 control households; **HH**: Household; **HHM**: Household member; **cOR**: Crude odds ratio; **aOR**: Adjusted odds ratio

### Cohort characteristics

**Fig. 1:**
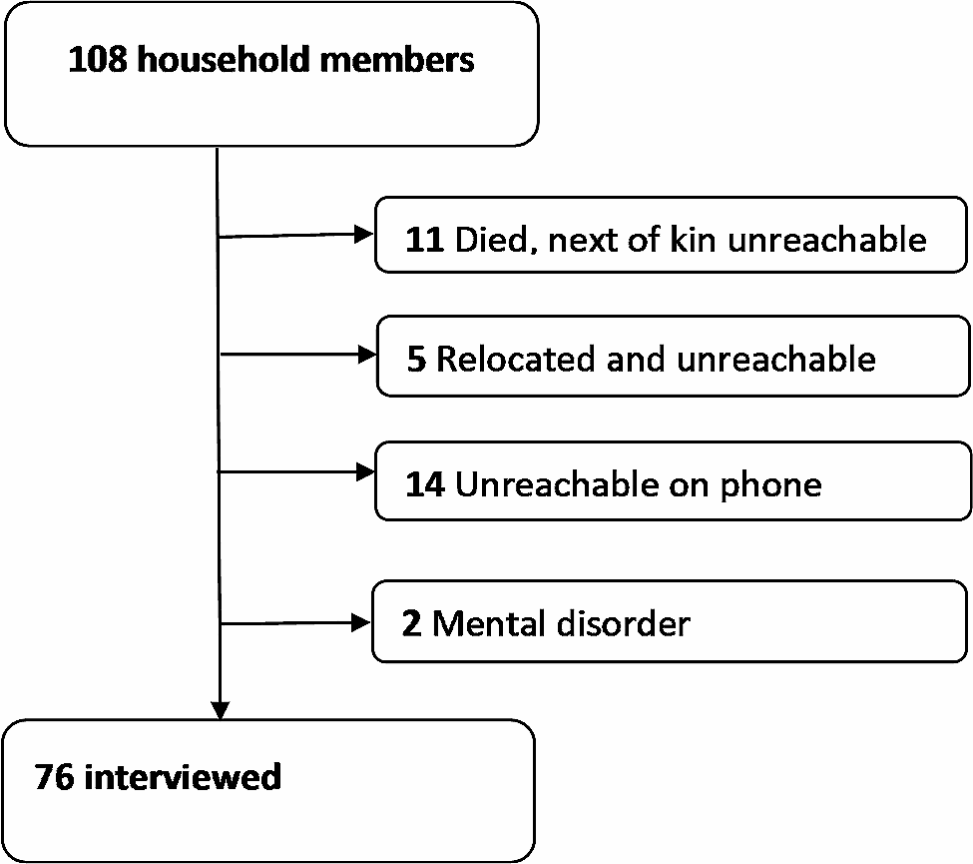
Persons enrolled in the cohort study of risk factors for SUDV infection, Uganda, 2022

From 19 case-households in the case-control study, we enrolled 76 of 108 total household members for the cohort study (Fig. 1).

Mean HHM age was 24 (± 17) years. Mean number of days from primary case-patient’s reported onset date to the HHM’s onset date was 8 (range, 1–20); median was 7 (IQR 4–10). A total of 44 (58%) HHM were tested for SUDV infection. Twenty-nine (38%) HHM overall who had illness onset ≤ 2 weeks from their last contact with the primary case-patient tested positive for SUDV infection (Table 2).

**Table 2:** Characteristics of household members (HHM) (*n* = 76) of primary SVD cases in Mubende and Kassanda districts, Uganda, 2022

Variable	Frequency	%
Age		
< 18	33	43
≥ 18	43	57
Mean age (± SD)	24 (± 17)	
**Sex**		
Male	30	39
Female	46	61
**Relationship of HHM to primary case-patient**		
Daughter/son	15	20
Mother/ father	18	24
Sibling	18	24
Husband/ wife	8	11
Aunt/ uncle	4	5
Grandchild	7	9
Other	6	8
**Occupation of HHM**		
Child or student	36	47
Other professions	40	53
**Highest education attained**		
None and primary	67	88
Secondary and above	9	12
**HHM developed signs/symptoms of SVD (self-reported)**		
Yes	32	42
No	44	58
**Days from primary case-patient illness onset to HHM onset**		
Median (IQR)	7 (4-10)	
**HHM tested for SUDV**		
Yes	44	58
No	32	42
**SUDV test result (among all household members)**		
Positive	29	38
Negative	47	62
**HHM had an underlying condition**		
Yes	8	11
No	68	89
**HHM interacted with primary case-patient during illness**		
Yes	63	83
No	13	17
**Household suspected primary case-patient had SVD**		
Yes	3	4
No	73	96
**HHM had gloves**		
Yes	2	3
No	74	97
**HHM had access to handwashing station with soap**		
Yes	60	79
No	16	21
**Frequency of handwashing** ^**a**^		
Less than half of the time	47	78
More than half the time/ all the time	13	22
**HHM tried to keep distance from primary case-patient** ^**2**^		
Yes	5	7
No	64	93
**HHM knew how to interact with primary case-patient safely** ^**2**^		
Yes	3	4
No	66	96
**HHM was given information on caring for primary case-patient safely** ^**b**^		
Yes	4	6
No	65	94

^**a**^*n*=60; ^**b**^*n*=69; SD: Standard deviation; IQR: Interquartile range; HH: Household; HHM: Household member; SVD: Sudan virus disease; SUDV: Sudan virus

By individual interaction (not mutually exclusive), infection rates were highest among those who had sexual intercourse with the primary case-patient during his or her illness (Table 3).

**Table 3:** Interactions between household members and primary case-patients in Mubende and Kassanda districts, Uganda, and subsequent positive test proportion, 2022 (*n* = 63)

Interaction	Total, *n*	SUDV +, *n*	(%)
Had sexual intercourse with primary case-patient	3	2	(67)
Shared bed with primary case-patient	37	22	(59)
Carried/ held primary case-patient	27	16	(59)
Bathed/ cleaned primary case-patient	31	18	(58)
Helped primary case-patient move around	21	12	(57)
Fed primary case-patient	27	15	(56)
Cleaned primary case-patient’s room	20	11	(55)
Changed primary case-patient’s beddings	35	19	(54)
Washed primary case-patient’s clothes	36	19	(53)
Breastfed primary case-patient	2	1	(50)
Removing primary case-patient’s dishes	47	23	(49)
Played with primary case-patient	13	6	(46)
Shared utensils with case-patient at meals	42	18	(43)
Rode on boda with primary case-patient	7	3	(43)
Sat with primary case-patient in same room	27	10	(37)
Exchanged money with primary case-patient	7	0	(0)

Among the 76 household members, 13 (17%) had no contact with the primary case-patient in their household; none of these 13 became ill. Four (5%) had minimal contact, of whom two became ill. Three (4%) had indirect contact, and one became ill. Fifty-six (74%) had direct contact, and 26 became ill (Table 4).

**Table 4:** SUDV positivity by level of contact among household members of primary case-patients in Mubende and Kassanda districts, Uganda, 2022 (*n* = 76). Interaction levels represent the maximum degree of interaction between the household member and primary case-patient

Interaction category	Total (col %)	SUDV + (row %)
	*n* (%)	*n* (%)
No contact	13 (17)	0 (0)
Minimal contact	4 (5)	2 (50)
Indirect contact	3 (4)	1 (33)
Direct contact	56 (74)	26 (46)

Household members who had direct contact with the primary case-patient in their households had a three-fold higher risk of contracting SUDV infection than those who had either no, or minimal or indirect contact only. Additionally, those who had direct and/or indirect contact had four times the risk of SUDV infection compared to those who had no contact and/or minimal contact (Table 5).

**Table 5:** Grouped interactions of household members of primary case-patients in Mubende and Kassanda districts, Uganda, 2022

Exposure (*n* = 76)	*n*	SUDV +	uRR^*^ (95% CI)	*P* value
**Any direct contact vs. (no contact, minimal contact, or indirect contact only)**				
No, minimal, or indirect contact	20	3	1	
Direct contact	56	26	3.1 (1.1–9.1)	0.042
**Any direct or indirect contact vs. (no contact or minimal contact only)**				
No contact or minimal contact	17	2	1	
Direct and indirect contact	59	27	3.9 (1.02-15)	0.047

^*^uRR – unadjusted risk ratio; SUDV: Sudan virus

In multivariable analysis, being aged ≥ 18 years (aRR_adj_=1.9, 95% CI: 1.01–3.7) and having had direct and/or indirect contact but not minimal contact with the primary case-patient (aRR_adj_=3.2, 95% CI: 1.1–9.7) increased the risk of SUDV infection among household members. Access to a handwashing facility decreased the risk of SUDV infection (aRR_adj_=0.52, 95% CI: 0.31–0.88) (Table 6).

### Risk factors for SUDV infection among HH members

**Table 6:** Risk factors for SVD among household contacts to primary case-patients in Mubende and Kassanda districts, Uganda, 2022

Exposure (*n* = 76)	*n*	SUDV +	uRR (95% CI)	aRR (95% CI)	*P* value
**Age**					
< 18	42	22	1	1	
≥ 18	34	8	2.1 (1.08–4.2)	1.9 (1.01–3.7)	**0.05**
**Access to handwashing facility**					
No	16	10	1	1	
Yes	60	19	0.51 (0.29–0.86)	0.52 (0.31–0.88)	**0.013**
**Sex**					
Male	30	11	1	1	
Female	46	18	1.07 (0.59–1.9)	1.1 (0.63–1.9)	0.69
**Highest education**					
None and primary	67	26	1	1	
Secondary and above	9	3	0.86 (0.33–2.3)	0.84 (0.35-2.0)	0.70
**Primary case-patient’s outcome**					
Died	24	12	1	1	
Recovered	52	17	0.65 (0.37–1.1)	0.8 (0.44–1.4)	0.38
Direct or indirect contact vs. (no contact or minimum contact) *					
No contact or minimal contact	17	2	1	1	
Direct or indirect contact	59	27	3.9 (1.02-15)	3.2 (1.1–9.7)	**0.004**

*Representing the maximum level of contact a household member had with a case-patient

uRR: unadjusted risk ratio; aRR: adjusted risk ratio; SUDV: Sudan virus

## Discussion

This study found multiple factors to be associated with SUDV infection among household members of confirmed cases during the 2022 outbreak in Uganda. Having a case-patient die increased odds of a household having secondary SUDV cases. Households that had more than one bedroom had lower odds of having secondary infections than those that had only one bedroom. Higher levels of contact with the case-patient, especially nursing care, shared sleeping space, and sexual contact were associated with increased infection risk among household members. Being an adult household member and not having access to a handwashing facility increased risk of infection.

Nearly 90% of primary case-patients in case-households died, compared to slightly more than half of those in control households. This finding is similar to those from two studies in Sierra Leone where index patient death was a risk factor for household transmission [19, 20]. The association between primary case-patient death and secondary cases among household members may be due to the increased infectiousness during advanced disease [6] as a result of an increase in viral load [21]. Death among patients with EBOD has been associated with delays to care [22], which may be correlated with a longer time at home and increased time to expose household members. Interestingly, in our study, we did not identify differences between case-households and control households in the amount of time the primary case-patient spent at home while ill, which may suggest that the primary case-patients in case-households faced more rapid disease progression than those in control households. However, we lacked sufficient clinical data to assess this possibility.

Households that had more than one bedroom had lower odds of having secondary infections than those that had only one bedroom. However, the odds of SUDV infection did not differ significantly by the total number of rooms in the household or by the ratio of household members to either total rooms or bedrooms. This suggests that while crowding itself might not have increased risk in our study, shared sleeping space was a specific risk. In support of this finding, assessment of individual (non-mutually exclusive) interactions showed that infection rates were highest among those who had sex with the primary case-patient (67%) followed by those who shared a bed with the primary case-patient (59%). Infection during sexual intercourse may have occurred due to the requisite physical intimacy of the act, or possibly through sexual transmission. Although sexual transmission has only been documented from survivors [23], the virus is known to be present in body fluids including semen and vaginal fluids, and this is a possible mechanism of transmission. However, it is likely that persons having sexual intercourse with a patient also had other (nonsexual) exposures to the patient, possibly later in the illness, that could have put them at risk. In addition, only three of the 37 household members sharing a bed with the primary case-patient reported sexual intercourse with the primary case-patient, and infection rates were similar between these two groups. This suggests that simple proximity during sleeping may suffice to transmit infection, either due to physical contact or fomite contact.

In agreement with other studies demonstrating that close physical contact — and specifically nursing care — is a strong risk factor for infection [5, 24], we found that bathing or cleaning a patient, carrying, helping to move around, playing with, feeding or breastfeeding a patient, and sharing utensils at meals with the primary case-patient all increased risk. In the absence of direct contact, risk of infection reduced greatly, and no household members without any contact developed infection; this is consistent with other studies [5, 24]. While fomites can serve as a source of ebolavirus infection, this study adds to the body of evidence that this is a less common infection pathway than direct contact [5, 6].

In the cohort study, adult household members (18 years or older) were more likely to contract SUDV infection than those younger than 18 years. This finding is consistent with those from two studies that revealed that children are usually less affected than adults during Ebola outbreaks [3, 5, 25]. In contrast, Fang et al. [26] found that children had higher odds of household infection than adults, although this finding was not statistically significant. A possible explanation for children being less affected could be their limited ability to provide nursing care to ill family members, reducing their chances of a high-risk exposure. However, children — especially those < 5 years of age — have a higher case-fatality rate than adults from EBOD [27–29], and measures should be instituted to protect them even when they do not provide direct care to a patient.

In our cohort study, access to a handwashing facility reduced risk of infection among household members by half. This is not surprising; ebolavirus infections are primarily transmitted through contact with contaminated body fluids [4–6]. Handwashing can reduce risk by supporting both the patient’s own hygiene as well as the hygiene of his or her caretakers. Inclusion of messaging that emphasizes hand hygiene in communities affected by EBOD continues to be important.

Our study had several limitations. First, due to varied incubation periods, it is possible that some household members within a single household may have been infected by a common source external to the household and had different onsets. For example, siblings caring for an ill mother living outside the home might have both acquired infection from the mother at a similar time or perhaps sequentially. The median reported serial interval between the primary and secondary cases in our study was 7 days, four days shorter than the reported serial interval for the outbreak overall [30], suggesting that this limitation may have applied to at least some of our secondary cases. This would have made the associations between primary and secondary cases in case-households appear stronger than they really are. Second, in some households (particularly when a case-patient had died), a proxy was interviewed. The proxy might not have had accurate knowledge of interactions that may have occurred between the primary case-patient and household members, or of specific dates. Recall bias might also have resulted in under-reporting of some interactions. Finally, some sensitive interactions, such as sexual contact, may have been under-reported and thus the risk they posed may not have fully been assessed.

## Conclusion

Direct contact, and particularly sharing sleeping space or providing nursing care to an EBOD patient, increased risk of infection among household members. Health messaging during EBOD outbreaks should emphasize the risk of both sleeping next to and providing nursing care for persons with symptoms consistent with EBOD, even early EBOD, until testing can rule out infection. However, we note that in the early stages of SUDV outbreak, direct contact of household members with primary case-patients may be inevitable. Often, before a diagnosis is made, unsuspecting household members have already been exposed to infectious primary case-patients. Implementation of risk assessments for household contacts may provide important data to justify closer monitoring of those considered to be at especially high-risk during contact tracing activities.

### Electronic supplementary material

Below is the link to the electronic supplementary material.

Supplementary Material 1

Supplementary Material 2

## Data Availability

The data upon which our findings are based belongs to the Uganda Ministry of Health and cannot be shared publicly for confidentiality reasons. However, it can be made available by the corresponding author with permission from the Ministry of Health Uganda, Division of Health Information and Uganda Public Health Fellowship Program.
