# Supplementary material for: Individual and household risk factors for Ebola disease among household contacts in Mubende and Kassanda districts, Uganda, 2022
Source: BMC Infect Dis. 2024 May 30;24:543. doi: 10.1186/s12879-024-09439-1 (PMC11138048; doi:10.1186/s12879-024-09439-1)
Supplement: Supplementary file 1 — Supplementary Material 1 [file 12879_2024_9439_MOESM1_ESM.docx]

**Questionnaire**

**Individual and household risk factors for Ebola disease among household contacts in Mubende and Kassanda districts, Uganda, 2022**

**Informed consent for Ebola Virus Disease Investigation**

Hello. My name is_________________. I work with the Ministry of Health. We are in the district to learn more about the Ebola outbreak how Ebola spread, and its impact on the community in Mubende and Kassanda Districts.

We would like to visit your home and ask you some questions about your household and your experience with Ebola. We will ask you a few different sets of questions. One will be about your household, one will be about your interactions at home with the patient or others at home, and one will be about how the outbreak has affected the mental well-being of you and your family members. The interviews will last approximately one hour per person.

There are no direct monetary benefits to you if you agree to participate in this interview. However, the information that you give us will help us to understand how we can support the community in the event of another Ebola outbreak. The information you share is strictly private and will only be shared by members of the investigation team.

Participation in this interview is voluntary, and you can stop the interview at any time. You do not have to answer any questions you don’t want to answer. Do you want to ask me anything about the information we are collecting or the survey?

Do you agree to participate?

Yes__________________ No_________________________________

**PART A: ELIGIBILITY QUESTIONS**

1. **Who was the first person to be ill in this household? ____________**
2. **Was this person at home for at least 24 hours while ill with symptoms of EVD?**
   1. **Yes 🡪 Continue**
   2. **No 🡪 Stop**
3. **What is this person’s C-number? |____|____|____|**
4. **During that time, did other people live in the household with this patient (regardless of whether or not they are family members)?**
   1. **Yes 🡪 Continue**
   2. **No 🡪 Stop**
5. **If yes, how many other people lived (slept overnight) in the household with this patient while he/she had symptoms of EVD? ______**
6. **Of the OTHER PEOPLE sleeping in the household with the first patient, did any others become ill with confirmed EVD?**
   1. **If YES, how many became ill with EVD <2 weeks after the last exposure to the primary case? _____ (list dropdown of names with case IDs)**
      1. **If >0, case-household**
      2. **If 0, control-household**

**GENERAL HOUSEHOLD INFORMATION**

***Fill this section in for case- and control-households***

GPS Coordinates:___________________ Longitude__________Latitude________________

District: __________Sub-county_______________ Village________________

1. Where is the household located?
2. Village (rural)
3. Town council/ Trading centre/ Municipality

**SECTION 1: HOUSEHOLD QUESTIONS FOR CASE AND CONTROL HHs**

1. What are the walls made of? (***Observe)***
2. Mud
3. Grass
4. Unburnt bricks
5. Plastered/ Burnt bricks
6. Concrete
7. Other, specify___________________
8. What is the home flooring made of? (***Observe)***
9. Mud/ Earth/ sand
10. Stone/concrete
11. Wood, bamboo, or palm
12. Tiles or cement
13. Other, specify____________
14. What is the roof material made of? (***Observe)***
15. Mud
16. Grass/Papyrus/Banana leaves
17. Zinc/Iron Sheets
18. Tiles
19. Other, specify: _______________
20. How many rooms does this home have (total)? __________
21. How many bedrooms does this home have (for sleeping)? ________
22. Does this home have (tick all that apply)
    1. Jerrycans for handwashing
    2. Piped water for handwashing
    3. Other handwashing setups, specify
23. Does the home have electricity? Yes No 🡪 Skip to 13
24. If yes, specify
25. Wired
26. Solar
27. Generator
28. Other, specify: ___________________
29. Does the home have a dustbin? ____________

**PART B: EXPOSURE FACTORS FOR THE PRIMARY CASE (FOR CASE AND CONTROL HHS)**

***Questions in this section should ONLY be asked to the primary case-patient/their proxy.***

***(Only those who had symptoms for atleast 3 days before evacuation, and live(d) with alteast one person)***

1. Who is answering the questions for the primary case-patient?
   1. Self (primary patient)
   2. Someone else, specify relationship
      1. Daughter/Son
      2. Mother/Father
      3. Sibling
      4. Husband/ wife
      5. Grandparent
      6. Aunt/uncle
      7. Grandchild
      8. Friend
      9. Other, specify: _________________
   3. Specify reason a proxy is answering for the primary case
      1. Primary case died
      2. Primary case not home
      3. Primary case too young/unable to answer
      4. Primary case does not want to answer
2. **Individual unique Identifier for primary case: |____| |____|____| (*check the list with c-numbers)***
3. What is/ was the age of the primary case in years? |____|____| (if <1, list 00)
4. Sex of the primary case
5. Male
6. Female
7. How many days was the primary case sick and sleeping at home? _____ days
8. Did the primary case have signs and symptoms? Yes____________No______
9. What signs and symptoms did the primary case have **while at home** (before being evacuated) (tick all that apply – ask one by one)?
10. Fever
11. Headache
12. Joint pains
13. Abdominal pain
14. Cough
15. General body weakness
16. Muscle pain
17. Hiccups
18. Vomiting
19. Haemoptysis
20. Diarrhoea
21. Conjunctivitis
22. Jaundice
23. Bleeding from the nose
24. Bleeding from the gums
25. Blood in urine
26. Blood in stool
27. Other (Specify)
28. What was the primary case’s clinical outcome?
29. Recovered
30. Died
31. Does/ did primary case have any underlying condition? **Yes No 🡪 Q 23**
32. If yes, Which one of these
33. Diabetes
34. Hypertension
35. TB
36. HIV
37. Other condition (specify)
38. DK
39. None
40. Did the primary case stay isolated at home (in a room by himself or herself) while ill at home before evacuation to ETU? **Yes No 🡪 Q 26**
41. Ask person about the isolation room, check all that apply)?
    - Has its own toilet facility
    - Has its own handwashing basin
    - Any other notes: ____________________________________
42. Did anyone at home suspect that the patient might have EVD when he/she became ill?
    1. Yes
    2. No
    3. DK
43. Did the household have discussions about how to protect themselves from the infection in the primary case before he/she was evacuated? Yes No DK
44. Did the primary case have information about how to self-isolate? **Yes No 🡪 Q** **28**
45. If yes, how was the information delivered?
    - 1. Verbal information (something told to them by someone else)
      2. Visual information (from a poster)
      3. Written information (something handed to the case)
46. Did the primary case understand how to self-isolate when he/she became ill?**Yes No DK**
47. Did the primary case have a single dedicated caretaker during his/her illness? **Yes No**
48. If not, how many people took care of him/her during their illness? _________
49. What type of care or interactions did household members provide while the primary case was ill (read individually)?
50. Breastfed primary case
51. Brought primary case food or water
52. Took away primary case dishes / cups
53. Shared meals or dishes
54. Changed primary case’s bedding
55. Did laundry for primary case
56. Cleaned room of primary case
57. Sat with case / played / talked to case inside the room
58. Helped primary case move around
59. Other, specify _______________________________
60. None of the household members took care of case while ill
61. Did the primary case share a mobile phone with other HH members during his/her illness?
62. Yes, specify who you shared with: ___________________
63. No
64. Did the primary case share a sleeping room with other HH members during his/her illness?
65. Yes, specify who: ___________________
66. No
67. Did the primary case share a bed with other HH members during his/her illness?
68. Yes, specify who: ___________________
69. No
70. Was there a dedicated toilet for the primary case used only during his/her illness?
71. Yes
72. No
73. Was there a dedicated handwashing station (even if just a jerrycan or tub) for the primary case’ use only during his/her illness?
74. Yes
75. No
76. How often did the primary case wash his/her hands during illness?
77. Several times a day
78. Once a day
79. Never / almost never
80. Did the primary case leave the home during his/her illness?
81. Yes, to go where? __________________
82. No
83. Did primary case or his/her family experience any stigma during illness before the primary case was evacuated to the ETU?
84. Yes, specify: ________________________________________
85. No

***************************************************************************

**PART C: EXPOSURES FOR ALL HOUSEHOLD MEMBERS OF PRIMARY CASE (FOR CASE-HOUSEHOLDS IN WHICH A SECONDARY CASE OCCURRED <2 WEEKS AFTER LAST EXPOSURE TO PRIMARY CASE)**

***The following questions should be asked of everyone in the household of a primary case. If household member is alive but not there, interview on phone. If dead, interview their proxy.***

1. **Primary case c-number: |____|____|____|**
2. **Individual unique Identifier: |____|____|____| -- |____|** (c-number of primary case, and then sequential numbers for each household member)
3. ***Include c-number if THIS HH member became a case* |____| |____|____|**
4. What is the age of the household member in years? |____|____|
5. Sex of this hh member **Male Female**
6. If female contact, was she pregnant when she lived with the primary case?
7. Yes
8. No
9. Relationship of this hh member to primary case
10. Daughter/Son
11. Mother/Father
12. Sibling
13. Husband/ wife
14. Grandparent
15. Aunt/uncle
16. Grandchild
17. Friend
18. Other, specify: _________________________
19. Occupation of HH member
    1. Farmer
    2. Teacher
    3. Trader / business person
    4. Transporter
    5. Healthcare worker
    6. Student
    7. Child (not student)
    8. Unemployed
    9. Other, specify: _________________
20. Highest education attainment
21. None
22. Primary
23. Secondary
24. Tertiary
25. Don’t know
26. Was this HH member ever tested for Ebola? **Yes No 🡪 Q**
27. If yes, what was the test result? **Positive Negative Don’t know**
28. What was the patient’s clinical outcome? **Recovered Died**
29. Did hh member develop symptoms of Ebola after the primary case’s illness (regardless of whether or not he/she was tested)? **Yes No** 🡪**QXXX**
30. **If yes, how long after the primary case developed symptoms did you become ill? ___ days**
31. If yes, which symptoms (select all that apply)?
32. Fever
33. Headache
34. Joint pains
35. Abdominal pain
36. Cough
37. General body weakness
38. Muscle pain
39. Hiccups
40. Vomiting
41. Haemoptysis
42. Diarrhoea
43. Conjunctivitis
44. Jaundice
45. Bleeding from the nose
46. Bleeding from the gums
47. Blood in urine
48. Blood in stool
49. Other (Specify)
50. Does HH member have any underlying condition? **Yes No 🡪 QXX**
    1. If yes, which one of these (all that apply)
51. Diabetes
52. Hypertension
53. TB
54. HIV
55. Other condition (specify)
56. None
    1. If HIV-positive, is contact on ART? Yes No
57. Did HH member interact with primary case during primary case illness? **Yes No 🡪 Q**
58. What type of interaction/care **did HH member provide** for primary case while at home?
59. Breastfed him/her (for mothers of cases)
60. Fed him/her
61. Shared plates, silverware, or glasses with the case during meals
62. Took away his/her dishes / cups
63. Slept with the case (shared a bed)
64. Changed his/her bedding
65. Washed his/her clothes
66. Bathed/ cleaned him/her
67. Carried/ held him/her
68. Played with him/her
69. Cleaned his/her room
70. Helped him/her move around
71. Sat with him/her / played / talked to him/her inside the same room
72. Rode on a boda with the case
73. Rode in the same car to somewhere with the case
74. Exchanged money with the case
75. Had sexual intercourse with the case
76. Other, specify _______________________________
77. Did you suspect that the patient might have EVD when he/she became ill?
    1. Yes
    2. No 🡪 Skip to Q52
    3. DK 🡪 Skip to Q52
78. Did you change your behavior in any way when you suspected that the primary case might have EVD to protect yourself? Yes No/DK 🡪 Skip to next section
79. In what ways did you change your behavior? ________________________________

***-----------------------IF HH MEMBER IS <5 YEARS, STOP HERE----------------------------***

1. Did HH member have gloves to wear during the primary case’s illness? Yes No
2. If yes, how often did HH member wear them while taking care of the primary case?
3. 100% of the time
4. More than half the time but less than all of the time
5. Less than half of the time
6. Never
7. I did not take care of the primary case
8. Did you change the gloves?
   1. Yes
   2. No
9. If yes, how often did you change them in a day?
   1. Frequently
   2. Sometimes
   3. Rarely/Never
10. Does HH member have access to a handwashing station with soap at this household? Yes No
11. If yes, how often did you wash your hands while interacting with the primary case?
12. 100% of the time
13. More than half the time but less than all of the time
14. Less than half of the time
15. Never
16. Did HH member try to keep distance from the primary case while the primary case was ill?
    1. Yes
    2. No
    3. DK
17. Did HH member know what to do to care for/interact with the primary case safely? **Yes No**
18. Did anyone provide HH member with information about how to care for the primary case safely? **Yes No**
19. If yes, where did they hear the information?
20. Local leaders (VHTs, LCs)
21. Radio
22. TV
23. Moving car adverts
24. Other (specify)
25. Does hh member have any comments or questions you would like to add?

________________________________________________________________________

*Thank you for answering our questions today…something about other people coming to your home with questions for different reasons. How to contact us.*

*****************************THE END*************************************
