# Supplementary material for: Individual and household risk factors for Ebola disease among household contacts in Mubende and Kassanda districts, Uganda, 2022
Source: BMC Infect Dis. 2024 May 30;24:543. doi: 10.1186/s12879-024-09439-1 (PMC11138048; doi:10.1186/s12879-024-09439-1)
Supplement: Supplementary file 2 — Supplementary Material 2 [file 12879_2024_9439_MOESM2_ESM.docx]

**Figure 1: Persons enrolled in the cohort study of risk factors for SUDV infection, Uganda, 2022**
